# The impact of clinical and population strategies on coronary heart disease mortality: an assessment of Rose’s big idea

**DOI:** 10.1186/s12889-021-12421-0

**Published:** 2022-01-06

**Authors:** Mohadeseh Ahmadi, Bruce Lanphear

**Affiliations:** Faculty of Health Sciences, Simon Fraser University, Burnaby, Canada

## Abstract

**Background:**

Coronary heart disease (CHD), the leading cause of death worldwide, has declined in many affluent countries but it continues to rise in industrializing countries.

**Objective:**

To quantify the relative contribution of the clinical and population strategies to the decline in CHD mortality in affluent countries.

**Design:**

Meta-analysis of cross-sectional and prospective studies.

**Data sources:**

PubMed and Web of Science from January 1, 1970 to December 31, 2019.

**Method:**

We combined and analyzed data from 22 cross-sectional and prospective studies, representing 500 million people, to quantify the relative decline in CHD mortality attributable to the clinical strategy and population strategy.

**Result:**

The population strategy accounted for 48% (range = 19 to 73%) of the decline in CHD deaths and the clinical strategy accounted for 42% (range = 25 to 56%), with moderate inconsistency of results across studies.

**Conclusion:**

Since 1970, a larger fraction of the decline in CHD deaths in industrialized countries was attributable to reduction in CHD risk factors than medical care. Population strategies, which are more cost-effective than clinical strategies, are under-utilized.

## Article summary

**Table:** 

Strengths and limitations of the study	
The studies in this meta-analysis relied on the IMPACT model that has been validated and calibrated against reliable data and replicated across different populations.	
The CHD IMPACT model is comprehensive: it includes CHD treatments and an extensive list of risk factors for CHD, with the notable exception of heavy metals and air pollution.	
The studies in this meta-analysis were constrained by available data and assumptions.	
The studies in the meta-analysis were based on industrialized countries; none were done in low-income, industrializing countries.	
We lacked national-level data to evaluate the hypothesis that the relative decline in CHD is attributed to countries’ investment in medical care and risk factor reduction.	

## Background

Coronary heart disease (CHD) is the leading cause of death globally [1]. In 2015, CHD accounted for 111 million (27%) of the 400 million cases of cardiovascular (CVD) worldwide [2]. CHD deaths are not uniform; rates of CHD have declined in affluent countries, such as the U.S and England, [3, 4] while they continue to increase in industrializing countries, such as China and India [5, 6]. The World Health Organization (WHO) projects that CHD will remain one of the top three causes of death globally, with nearly 9.3 million deaths annually in 2030 [1]. CHD is costly. In 2015, the CHD-associated financial burden in the U.S amounted to $188 billion; by 2035, the costs of CHD in the U.S are projected to exceed $366 billion [7]. In the United Kingdom (UK), CHD-associated costs exceed 7 billion EU annually [8]. In 2010, the costs of CHD in China were $17 US billion [9]. CHD thus poses a tremendous burden on countries and their economy and medical care systems.

Two distinct and complementary strategies exist to control CHD: the low-risk or population strategy and the high-risk or clinical strategy. In a landmark article, published in 1981, Geoffrey Rose argued that the population or “mass” strategy was more effective than the clinical strategy [10]. The population strategy, which is focused on the health of the entire community, attempts to shift downward the distribution of risk factors, like smoking or hypertension. In contrast, the clinical strategy targets a smaller fraction of the high-risk population. In 1993, Albert Hoffman, in a commentary titled, *Rose’s Big Idea*, wrote that there is a “general lack of data to support [Rose’s] view that preventive measures directed towards the whole population will not only in theory but also in practice prevent disease” [11]. What can we say now, forty years after Rose’s 1981 landmark article? Does the population strategy (risk factor reduction) or the clinical strategy (medical care) contribute to a larger reduction in CHD deaths?

The purpose of this article was to examine the relative contribution of population strategies and clinical strategies to the decline in CHD mortality. We conducted a meta-analysis of published studies to quantify the relative contribution of medical care and risk factor reduction on CHD mortality decline from January 1, 1970 to December 31, 2019.

## Method

**Fig. 1:**
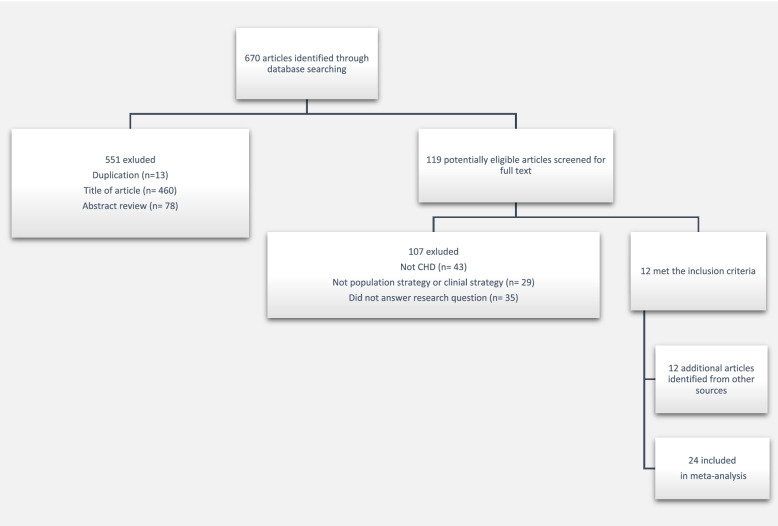
Flow chart of the literature search and screening process

We employed the PRISMA (Preferred Reporting Items for Systematic Reviews and Meta-Analyses) guidelines to conduct this meta-analysis. We conducted a comprehensive search for original articles in PubMed and Web of Science published from January 1, 1970 to December 31, 2019 using the primary search terms prevention strategies (population-based or clinical-based) and outcome variable (CHD) (Fig. 1). We did not specify any other outcomes. We also identified studies using the references of identified articles.

### Data selection process

We conducted a search in PubMed using the search string: (“Coronary Disease/mortality”[MeSH Terms] OR “coronary mortality” OR “coronary heart disease mortality”) AND ((primary prevention [MeSH Terms]) OR (secondary prevention [MeSH Terms])). This search generated 340 results. We also conducted a search in the Web of Science using the search string: ((CHD OR “coronary heart disease”) AND (“secondary prevention” OR population-based OR community-based OR low-risk) AND (“primary prevention” OR clinically based OR clinic-based OR clinical-based OR high risk) AND mortality AND (“risk factor” OR “risk reduction”)). It generated 324 results.

### Inclusion and exclusion criteria

We included studies that met the following criteria: based on English language; provided quantitative results; population samples from the general population (both healthy individuals and established cases of CHD); and reported reduction in CHD mortality for both strategies.

### Selection of studies for inclusion in the meta-analyses

The first author (MA) performed extensive search strategies in PubMed and Web of Science, identified, and subsequently screened titles and abstracts for eligibility against the inclusion criteria. To be included in the present meta-analyses, the first author then read and evaluated full text of each selected article for overall scientific. The other author (BL) further reviewed and verified the work of the first author and the extracted information.

### Methods for quality assessment

We used the internationally recognized GRADE framework to assess the quality of evidence presented in the studies included in the meta-analysis [12]. The quality of evidence was rated as “high”, “moderate”, “low”, or “very low” based on six assessment criteria, namely, indirectness, inconsistency, publication bias, magnitude of effect, risk of bias, and confounding. The grade or quality of evidence rating began at “high” for all the studies included in meta-analysis and subsequently downgraded if evidence in any of the six assessment criteria mentioned above could be determined.

### Data synthesis

The meta-analysis was conducted utilizing R software. *I*^*2*^ statistics was used to measure the level of heterogeneity regarding the reported proportion of decline in CHD mortality attributed to clinical or population across studies included in the meta-analysis. The levels of 25, 50 and 75 represented low, moderate, and high level of inconsistency, respectively [13].

The IMPACT model was used by all the studied in the meta-analysis to quantify the relative contribution of clinical and population strategies to decline in CHD mortality. This model is a mortality model that can calculate the number of CHD deaths prevented by each risk factor change and by each treatment intervention. To calculate the number of CHD deaths postponed by risk factor changes, two approaches were used. In the first approach, contribution of continuous variables such as blood pressure, BMI, and cholesterol level were measured. For this type of variables, the model used β coefficient obtained from meta-analyses and cohort studies. The β coefficient measures the relationship between changes in population level risk factor and the reduction in CHD mortality attributable to that change. To calculate the decline in CHD deaths from each continuous risk factors type, the product of the β coefficient, reduction in risk factor level from the baseline year, and CHD mortality in baseline year were calculated.

For categorical variables such as physical inactivity, smoking, and diabetes, the model used another measure known as population attributable risk factor (PAR) which includes the prevalence of the risk factor and relative risk of death from CHD attributable to that risk factor. To quantify the number of CHD deaths postponed from categorical risk factor types, the model calculated the product of the number of CHD deaths in baseline year and the difference between PAR in base year and that in the year of comparison.

$$\mathrm{PAR}=\frac{Prevalence\ast (Relative\ Risk- 1)}{1+\left[ Prevalence\ast \left( Relative\ Risk- 1\right)\right]}$$

Furthermore, to calculate the number of CHD deaths prevented from each treatment intervention, the model calculated the product of the age-specific case fatality rate, the number of subjects in that treatment group, the proportion of subjects receiving that treatment, and the treatment efficacy reported in meta-analyses. For the clinical strategy, the intervention included in the studies were thrombolysis, aspirin use, ACE inhibitors, β blockers, and so on. For the population strategy, reduction levels in prevalence of diabetes, smoking, physical inactivity, as well as reductions in blood pressure, obesity, and cholesterol level reported from official statistics were included.

### Meta-analysis/forest plots

In the studies included in the meta-analysis results were reported as proportion of CHD mortality decline due to population strategy and proportion of CHD mortality decline due to clinical strategy. The results from each study were combined to calculate an overall pooled proportion for the relative contribution of population and clinical strategies to CHD mortality reduction. For visual interpretation, we constructed forest plots. Pooled proportions, 95% confidence intervals, and forest plots were constructed using metaprop package in R. We employed a random effect model because we assumed that the effect size varies from one study to the next study. The statistical heterogeneity of 100% that resulted was primarily due to varying level of investments made into each strategy by each country, but also due to differences in study populations (such as age of subjects), and duration of each study.

#### Patient involvement

No patients were involved in the development of the research question, the design or implementation of this study, nor were they involved in the recruitment to or conduct of the study. We have no plans to disseminate the results to study participants.

## Results

### Study level characteristics

We identified 22 studies that evaluated the relative contribution of clinical and population strategies to the reduction in CHD mortality, representing 500 million people (Table 1). Seventeen of the studies were based in European countries, three in North America, one in Asia, and one in Palestine. The duration of these studies ranged from 10 to 25 years.

**Table 1:** Summary of the studies included in the meta-analysis and deaths attributed to clinical and population strategies

Author (Publication year)	Country	Total CHD deaths averted	Years Studied	Age of Participants	Main outcome		
					CHD deaths prevented or postponed		
					Population Strategy	Clinical Strategy	Unexplained
Unal et al. (2005) [14]	England & Wales	61,747	1981–2000	25–84 years	53%	38%	9%
Bennett et al. (2006) [15]	Ireland	3765	1985–2000	25–84 years	48%	44%	8%
Capewell et al. (2000) [16]	New Zealand	671	1982–1993	Entire population of 996,000 of central Auckland, New Zealand	54%	46%	0%
Björck et al. (2009) [17]	Sweden	13,180	1986–2002	25–84	55%	36%	9%
Laatikainen et al. (2005) [18]	Finland	373	1982–1997	35–64 years	53%	26%	21%
Ford et al. (2007) [19]	United States	341,745	1980–2000	25–84 years	44%	47%	9%
Unal et al. (2013) [20]	Turkey	35,720	1995–2008	35–84 years	42%	47%	11%
Palmieri et al. (2010) [21]	Italy	42,930	1980–2000	25–84 years	55%	40%	5%
Wijeysundera et al. (2010) [22]	Ontario, Canada	7585	1994–2005	25–84 years	48%	43%	9%
Bandosz et al. (2012) [23]	Poland	26,200	1991–2005	25–74 years	54%	37%	9%
Hotchkiss et al.(2014) [24]	Scotland	5770	2000–2010	> 25 years	39%	43%	18%
Flores-Mateo et al. (2011) [25]	Spain	8530	1988–2005	35–74 years	50%	47%	3%
Abu-Rmeileh et al. (2012) [26]	West Bank	125	1998–2009	25–75 years	66%	29%	5%
Bajekal et al. (2012) [27]	England & Wales	38,000	2000–2007	> 25 years	34%	52%	14%
Bruthans et al. (2012) [28]	Czech Republic	12,080	1985–2007	25–74 years	52%	43%	5%
Hughes et al. (2012) [29]	Northern Ireland	3180	1987–2007	25–84 years	60%	35%	5%
Pereira et al. (2013) [30]	Portugal	3760	1995–2008	25–84 years	42%	50%	8%
Aspelund et al. (2010) [31]	Iceland	295	1981–2006	25–74 years	73%	25%	2%
Kabir et al. (2013) [32]	Republic of Ireland	6450	1985–2006	25–84 years	48%	40%	12%
Soshiro et al. (2019) [33]	Japan	75,700	1980–2012	35–84 years	35%	56%	9%
Sobers et al. (2019) [34]	Barbados	139	1990–2012	> 25 years	19%	56%	25%
Marek et al. (2018) [35]	Slovak Republic	1820	1993–2008	25–74 years	41%	50%	9%

### The quality of the included studies

We evaluated the quality of evidence of the studies in the meta-analysis using the GRADE framework. The grade we assigned to each study were essentially the same because they all used the IMPACT model. All 22 studies were found to be “moderate” quality. We downgraded the studies for two domains: outcome assessment and confounding. For outcome assessment, the studies analyzed the potential effect of multiple treatment in an individual while the potential effect of both risk factor reduction and medical treatment was not considered. In addition, while the analyses accounted for two important confounders: age and sex, they did not consider socioeconomic differences.

### Meta-analyses

**Fig. 2:**
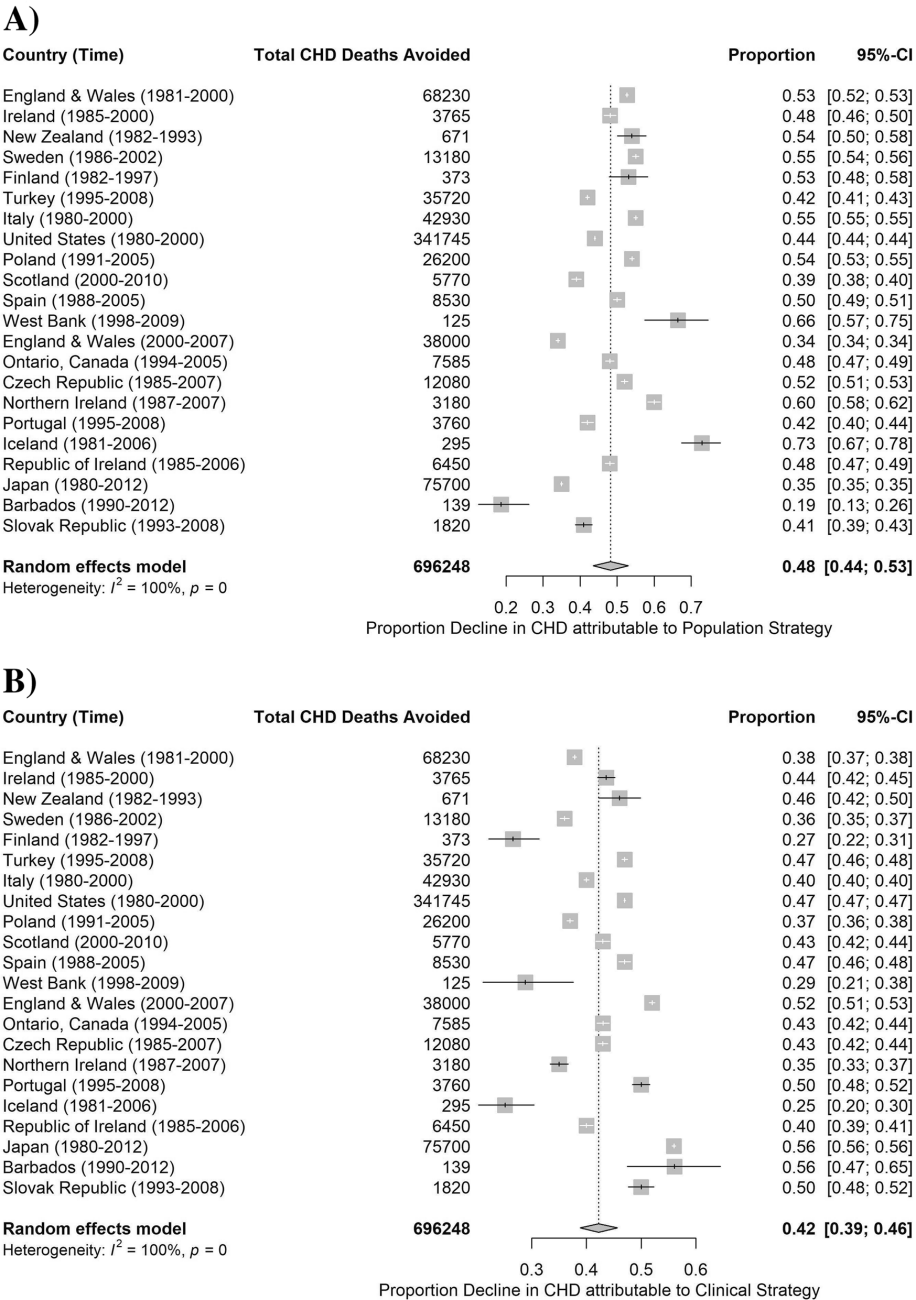
Forest plot showing stratified meta-analysis of CHD mortality decline attributable to population strategy (**A**) and clinical strategy (**B**). Proportions are shown as squares and 95% CI as horizontal lines. Heterogeneity, represented by *I* ^2^, explains the level of inconsistency between studies included in the meta-analysis

Fifteen of the 22 studies attributed a larger proportion of the mortality decline to the population strategy; seven studies attributed a larger proportion to the clinical strategy. The weighted proportion decline in CHD attributable to population strategy and clinical strategy was 48 and 42%, respectively (Fig. 2). The decline in CHD deaths attributed to the population strategy ranged from 19 to 73% whereas the decline in deaths attributed to the clinical strategy ranged from 25 to 56%. The unexplained proportion across the studies was 9%. The studies exhibited a considerable level of inconsistency, as shown by *I* ^2^ values of 100% for both population strategies and clinical strategies.

### Comparison between clinical strategy and population strategy

Population strategies led to a greater reduction in CHD deaths in most countries. In Finland, for example, CHD mortality declined by 63% from 1982 to 1997 [18]. Finland’s experience, however, was unique. To control the growing CHD burden in Finland, the North Karelia project was launched in 1971 [36]. The aim of the project was to implement community-wide interventions to control blood pressure, serum cholesterol, and smoking. After the first five years, the intervention was expanded to the rest of Finland [36]. Collectively, these reductions in risk factors contributed almost two-thirds of CHD mortality decline observed in Finland [36, 37]. In contrast, clinical strategies contributed more than population strategies in seven countries: the US, Turkey, Portugal, Scotland, Japan, Barbados, and Slovak Republic (Table 1). In the United States, for example, CHD mortality declined by about 40% between 1980 and 2000, equivalent to 341,745 deaths averted over the 20-year period [19]. The clinical strategy reduced CHD deaths by 47% and the population strategy reduce CHD deaths by 44% [19].

## Discussion

We conducted a meta-analysis of 22 cross-sectional and prospective studies conducted between 1970 to 2019 to quantify the relative contribution of the population and clinical strategies to the decline in CHD mortality. We found that of 48% the CHD decline was attributable to population strategies and 42% was due to clinical strategies. This finding, which supports Rose’s Big Idea, indicates that population strategies led to a larger reduction in CHD deaths than clinical strategies. Moreover, while we do not have country specific data on investments in population and clinical strategies, many countries spend the majority of their health dollars on medical care even though the population strategy is more cost-effective [38–40].

In his landmark article, Rose observed that a larger burden of disease will inevitably arise from the larger fraction of people who are at low-to-moderate risk than the smaller fraction of high-risk people [41–43]. The mass population strategy, in turn, greatly reduces the demand for expensive treatment and inevitable side effects of surgical procedures or pharmaceuticals. Conversely, a strategy focused on high-risk individuals, such as personalized precision medicine, will inevitably fail to prevent most cases of a disease or death [42]. Yet, as Rose noted, this led to a paradox: population strategies that bring large benefits to the entire community offer little to each participating individual [42].

Many people who might benefit from a medical or surgical intervention do not receive it. In 1981, Rose wrote, “In practice, however, treatment is not completely effective, all cases are not detected, and the people who are detected will often not take the treatment” [10]. Smith and his colleagues showed that about 50% of hypertensive population in Scotland went undetected [44]. Further, of those detected, almost half did not receive treatment and, of those who were treated, about half were not controlled properly [44]. Similarly, data from Sweden found that about half of people who had hypertension were aware of it and, of those, only two-thirds received medication [45].

Trends in CHD deaths vary widely by the level of industrialization. Over the past four or five decades, rates of CHD have declined in regulated, post-industrial countries, like the U.S and England [3, 4], whereas they increased in industrializing countries, such as China and India [5, 6]. In Mexico, for example, CHD mortality increased by 34% in men and 23% in female from 2000 and 2012, representing a total of 9370 additional CHD deaths in 2012. Approximately 71% of the increase in CHD mortality was attributed to rise in risk factors, while only about 42% of the deaths were potentially prevented as the result of advancement in medical therapies [46]. Similarly, CHD mortality increased by 64% in Syria between 1996 and 2006, where rise in CHD risk factors accounted for almost 81% of the increase, and suboptimal investment in treatment could only postpone 34% of it [47]*.*

Unfortunately, access to medical care is not widely available in many industrializing countries. Of the 30% of people in India with hypertension, for example, only one-third received optimal treatment [48]. Moreover, even with access to medical technology, the decline in CHD mortality achieved by coronary artery bypass graft surgery or stents – the most common surgical procedure in the US – are modest [49, 50]. These data further confirm that population strategies are crucial to control CHD in less affluent, industrializing countries. Although additional studies in developing countries have been conducted, they could not be included in this meta-analysis as they did not meet the inclusion criteria for language requirement.

Population strategies are especially effective for risk factors that are widely dispersed in the general population, like toxic metals and pollutants. Toxic metals, such as lead and arsenic, and airborne pollutants play a leading, if largely ignored role in CHD mortality [51, 52]. In a meta-analysis of 35 studies involving 348,259 study participants, Chowdhury et al. found that lead, arsenic and cadmium were associated with an increased risk of CHD [53]. Similarly, a population-based Chinese cohort study (hazard ratio = 1.43) [54] and a meta-analysis of 11 European cohorts (hazard ratio = 1.13) [55] established an increased risk of coronary heart disease with particulate air pollutants. Collectively, these data indicate that the contributions of toxic metal and air pollutants to CHD mortality have been greatly underestimated [53, 56–58]. Importantly, no apparent threshold exists for the risk of widespread exposures to lead and airborne pollution [53, 59].

If population strategies are so effective, why do we spend so little on them? [10] In the US, about 95% of all health dollars were spent on medical care; only 4% was spent to prevent disease [60]. Similarly, few research dollars were invested in prevention. From 2008 to 2019, the National Institute of Health funded 10,841 research projects to study coronary heart disease at a cost of $5 billion. Most funds flowed to laboratory or clinical research; only 3 % was spent on population studies [61]. Mass strategies led to a larger reduction in deaths from coronary heart disease than medical care, but they are grossly underfunded [38].

This meta-analysis has strengths and limitations. All the studies in this meta-analysis relied on the IMPACT model that is constrained by available data and assumptions. For example, because these studies did not incorporate key drivers of CHD, such as lead and air pollution – which declined in lockstep with CHD deaths over the past 50 years – they underestimated the impact of the population strategy [56, 57, 62]. Second, all the studies were based in affluent countries; none were done in lower income, industrializing countries. This meta-analysis relied on a modest number of studies, which may explain the observed heterogeneity of the results.

We found a wide range in the fractions attributed to both strategies. The decline in CHD attributed to either strategy is likely a reflection of several factors, such as countries’ investment in medical care and risk factor reduction, but we lacked national data to evaluate this hypothesis [39]. Finally, the risk factors included in the various studies were not always the same. All but one study included smoking prevalence, cholesterol level, blood pressure, obesity measures, diabetes, and physical inactivity as risk factors whereas three studies also measured mean level of fruit and vegetable consumption.

The IMPACT model has several strengths that makes it particularly appropriate for CHD modelling studies. The IMPACT model has been validated and calibrated against reliable data, and replicated across different population [63]. The estimated fall in CHD deaths are compared to observed fall in CHD mortality during the same period, often stratified by age and sex [63]. Lastly and most importantly, the CHD IMPACT model is comprehensive. It includes all CHD treatments and, except for heavy metals and air pollution, considers a comprehensive list of risk factors for CHD. Other models either fail to consider all treatments or only include a selected number of possible risk factors [63].

## Conclusion

Consistent with Rose’s *Big Idea*, we found that mass strategies targeting low and moderate-risk individuals results in a larger reduction in CHD mortality than providing expensive medical care to high-risk individuals. Moreover, the benefits of population strategies were underestimated because key drivers of the coronary heart disease epidemic, like heavy metals and air pollution, were not incorporated into these models. Clinical strategies will remain a critical safety net to treat symptoms of those who are already sick, but the goal should be to reduce risk factors on a population-level to quench the coronary heart disease pandemic.

## Data Availability

All data used in the study are available in published articles.
